# Duration, numerosity and length processing in healthy ageing and Parkinson’s disease

**DOI:** 10.1007/s10433-024-00807-z

**Published:** 2024-04-24

**Authors:** Z. Romeo, S. Dolfi, M. D’Amelio, G. Mioni

**Affiliations:** 1 Department of General Psychology, University of Padova, Via Venezia, 8, 35131 Padua, Italy; 2 Department of Developmental Psychology and Socialization, University of Padova, Padua, Italy; 3 University of Palermo, Palermo, Italy; 4 Neuroscience Institute, National Research Council (CNR), Padua, Italy

**Keywords:** Duration perception, Numerical perception, Length perception, Parkinson’s disease, Ageing

## Abstract

People constantly process temporal, numerical, and length information in everyday activities and interactions with the environment. However, it is unclear whether quantity perception changes during ageing. Previous studies have provided heterogeneous results, sometimes showing an age-related effect on a particular quantity, and other times reporting no differences between young and elderly samples. However, three dimensions were never compared within the same study. Here, we conducted two experiments with the aim of investigating the processing of duration, numerosity and length in both healthy and pathological ageing. The experimental paradigm consisted of three bisection tasks in which participants were asked to judge whether the presented stimulus (i.e. a time interval, a group of dots, or a line) was more similar to the short/few or long/many standards. The first study recruited healthy young and elderly participants, while the second recruited healthy elderly participants and patients with Parkinson’s disease, a clinical condition commonly associated with temporal impairments. The results of both experiments showed that discrimination precision differed between domains in all groups, with higher precision in the numerosity task and lower sensitivity in judging duration. Furthermore, while discrimination abilities were affected in healthy elderly and, even more so, in Parkinson’s disease group, no domain-specific impairments emerged. According to our research, reduced discrimination precision might be explained by an alteration of a single system for all quantities or by an age-related general cognitive decline.

**Supplementary Information:**

The online version contains supplementary material available at 10.1007/s10433-024-00807-z.

## Introduction

The processing of numerical, temporal and spatial information is essential in everyday life, and it supports basic activities such as selecting the shortest queue, bringing enough reusable bags at the supermarket, or brushing our teeth for the right amount of time. This fundamental ability has been the focus of several studies with the aim of clarifying how our brain can discriminate quantities and integrate different domains (Cappelletti et al. 2009; Hamamouche and Cordes 2019; Walsh 2003). The present study investigates the perception of time, numerosity and space in healthy or pathological elderly cohorts, with particular emphasis on Parkinson’s disease (PD).

For long time, researchers questioned about the existence of a unique system, rather than of distinct representations, for different types of dimensions (i.e. duration, numerosity and length) (Dehaene and Brannon 2010; Dormal and Pesenti 2012). One of the first hypotheses of a common magnitude system was the accumulator model by Meck and Church, who proposed that numerical and temporal information is represented in a similar manner through a common mechanism (i.e. an internal accumulator), ﻿that counts the impulses produced by a generator (Meck and Church 1983) each time an event or an object is encountered. ﻿More recently, the ATOM theory (A Theory of Magnitude, Walsh 2003) expanded the idea of a single representational system underlying time, number and space, suggesting the presence of a shared mechanism due to the need to integrate different dimensions to produce appropriate action responses. The ATOM theory also includes the space dimension in its model and proposes a partially shared common metric among time, number and space, together with dimension-specific processes (Walsh 2003). This hypothesis is supported by several studies that provided convergent results pointing to the parietal cortex as a common neural substrate involved in time, number and space processing (Dormal and Pesenti 2012; Hubbard et al. 2005). In particular, neuroimaging data showed that partially overlapping regions around the intraparietal sulcus are activated during the processing of different dimensions, leading to the suggestion that the parietal cortex represents the key brain region for quantities’ representation, as part of a larger network including the prefrontal cortex (Bueti and Walsh 2009; Dormal and Pesenti 2012). However, independent activation in response to time, number and space information has also been reported (for a review, see Dormal and Pesenti 2012; Sokolowski et al. 2017). ﻿For example, transcranial magnetic stimulation and neuropsychological data revealed a double dissociation between numerosity and duration processing (Cappelletti et al. 2009, 2011), which suggests the existence of partially distinct mechanisms. Behavioural evidence is also inconclusive, with mixed results on the similarity of the sensitivity thresholds of spatial magnitudes (e.g. area, length) and numerosity (DeWind and Brannon 2012; Leibovich and Henik 2014; Tibber et al. 2012). The discussion about the existence of distinct *vs.* common representation systems remains thus open and, although a large amount of behavioural and neurofunctional data have been provided, ﻿it is unclear whether duration, number and space processing are based on a common representation or supported by independent systems.

An interesting line of research to address this question focuses on how discrimination skills of temporal, numerical and spatial quantities evolve across the lifespan. A study by Droit-Volet and colleagues (2008) investigated the differences and the similarities in the discrimination of duration, numerosity and length, in children (5 and 8 years old) and adults. All groups showed flatter psychometric functions during non-sequential presentation and the sensitivity for time was lower than number and length, while the latter two dimensions showed similar patterns. While 5-year-old participants showed greater sensitivity to numerosity than duration or length, during sequential presentation no differences emerged between duration, numerosity and length in 8-year-old children and adults. The authors concluded that time, number, and space are part of a generalized magnitude system present from birth (Droit-Volet et al. 2008). However, other cross-sectional studies (from 3 to 11 years of age and adulthood) investigating discrimination abilities across different quantities showed a progressive differentiation between magnitude domains, with a steeper improvement in the discrimination of area and length, a slower growth in temporal magnitudes, and an intermediate trajectory for numerosity (Odic 2018; Odic et al. 2013). Recently, it has been shown that the interplay between time, number and space emerges very early in humans: neonates of a few days associate number and duration information to spatial length when these dimensions vary in the same direction (concomitant increase/decrease), but not in opposite directions (De Hevia et al. 2014). This suggests that before language acquisition or other kind of experiences, newborns can establish a connection between time, number, and space, demonstrating to be sensitive to the common structure of these magnitudes (De Hevia et al. 2014; Hamamouche and Cordes 2019; Walsh 2003).

On the other hand, less is known about discrimination processing during ageing or specific neurological conditions. An important aspect to clarify is whether magnitude processing is sensitive to ageing, due to a decline of a specific system as happens for other functions or, alternatively, whether the age-related decline is the result of deterioration of other cognitive processes involved in magnitude discrimination (Cappelletti et al. 2014; Halberda et al., 2012).

Research on elderly samples investigated the processing of a specific quantity (i.e. duration or numerosity), and never the comparison between the three dimensions (e.g. Capizzi et al. 2022; Cappelletti et al. 2014). Most of the studies have been conducted investigating temporal abilities in ageing and showed lower temporal abilities and higher variability in old compared to younger participants (Block et al. 1998; Wearden 2005). However, the strength of temporal impairment in older adults depended on the temporal task as well as the temporal range used. More recently, it has been suggested that a deficit of cognitive control functions, rather than of temporal processing, affects temporal abilities during ageing (Capizzi et al. 2022). Capizzi et al. (2022) evaluated temporal abilities in healthy and pathological elderly participants considering general cognitive decline with the Mini-Mental State Examination (MMSE). The results showed a flatter psychometric curve with decreasing MMSE scores and increasing age, while no shift of the psychometric function was observed, indicating that lower temporal abilities observed were mainly caused by a deficit at the level of cognitive control functions rather than of altered temporal processing per se (see also, Turgeon et al. 2016). Similarly, it has been proposed that a decline of the inhibitory processes could be responsible for a lower performance in numerosity discrimination tasks in elderly compared to young participants (Cappelletti et al. 2014; Norris et al. 2015). Length processing during ageing was less investigated. Few (and not recent) evidence showed that the ability to estimate the length of a line remains stable over an extended age range (Verrillo 1981). Also, it has been shown that time and space processing were equally accurate in younger and older participants (Lambrechts et al. 2013).

Notably, the lack of numerical, temporal and length data in the same sample does not allow to determine whether: (a) ageing is associated with a global cognitive impairment that, in turn, affects magnitude processing; (b) ageing is associated with a domain-specific deficit; c) healthy ageing does not affect discrimination abilities.

With respect to pathological ageing, there is evidence that particular neurological conditions (i.e. Parkinson’s disease) can affect the performance of specific discrimination tasks. Although the results are quite heterogeneous, PD patients commonly report deficits in tasks requiring temporal estimation (Koch et al. 2008; Malapani et al. 2002; Smith et al. 2007). Dormal et al. (2012) compared PD patients, healthy elderly, and healthy young participants in a numerosity and a duration task. The results confirmed an effect of ageing on duration comparison: elderly participants reported significantly more errors compared to the younger group. Interestingly, while the three groups did not show difference in the numerosity task, the performance of PD patients on the duration task was less accurate than both young and elderly participants (Dormal et al. 2012), indicating a specific impairment in the temporal domain.

The present research aims to investigate the processing of duration, numerosity and length in healthy and pathological elderly groups. To this end, we conducted two studies in which discrimination tasks were administered to healthy young and elderly participants (Study 1) and to healthy elderly participants and PD patients (Study 2). The paradigm consists of three bisection tasks in which participants were asked to judge whether the stimulus presented (i.e. a temporal interval, a group of dots or a line) was more similar to the short/few or to the long/many standards. The main goal is to understand whether elderly participants show impairments in the discrimination of a specific kind of magnitude or in all of them. In addition, this study also proposes to compare the performance of non-pathological elderly participants with PD patients, ﻿known to suffer from impairments in duration processing. To the best of our knowledge, this is the first research work that measures duration, numerosity and length discrimination performances in healthy young, healthy elderly participants and in a clinical population.

## Study 1

### Method

#### Participants

Thirty-four younger (mean age = 22.7 (2.26) years; level of education = 15.4 (1.31) years; female = 23) and 34 older (mean age = 71.8 (4.81) years; level of education = 13.7 (4.52) years; female = 20) adults participated in the present study. Exclusionary criteria for all participants included psychological, psychiatric, and neuropsychological disorders. For older adults, we used the Montreal Cognitive Assessment (MoCA, (Santangelo et al. 2015) to exclude participants with possible dementia or severe cognitive impairment as defined by MOCA scores below the cut-off of 18 (mean MoCA = 26.8 (1.49)).

#### Bisection task

**Fig. 1:**
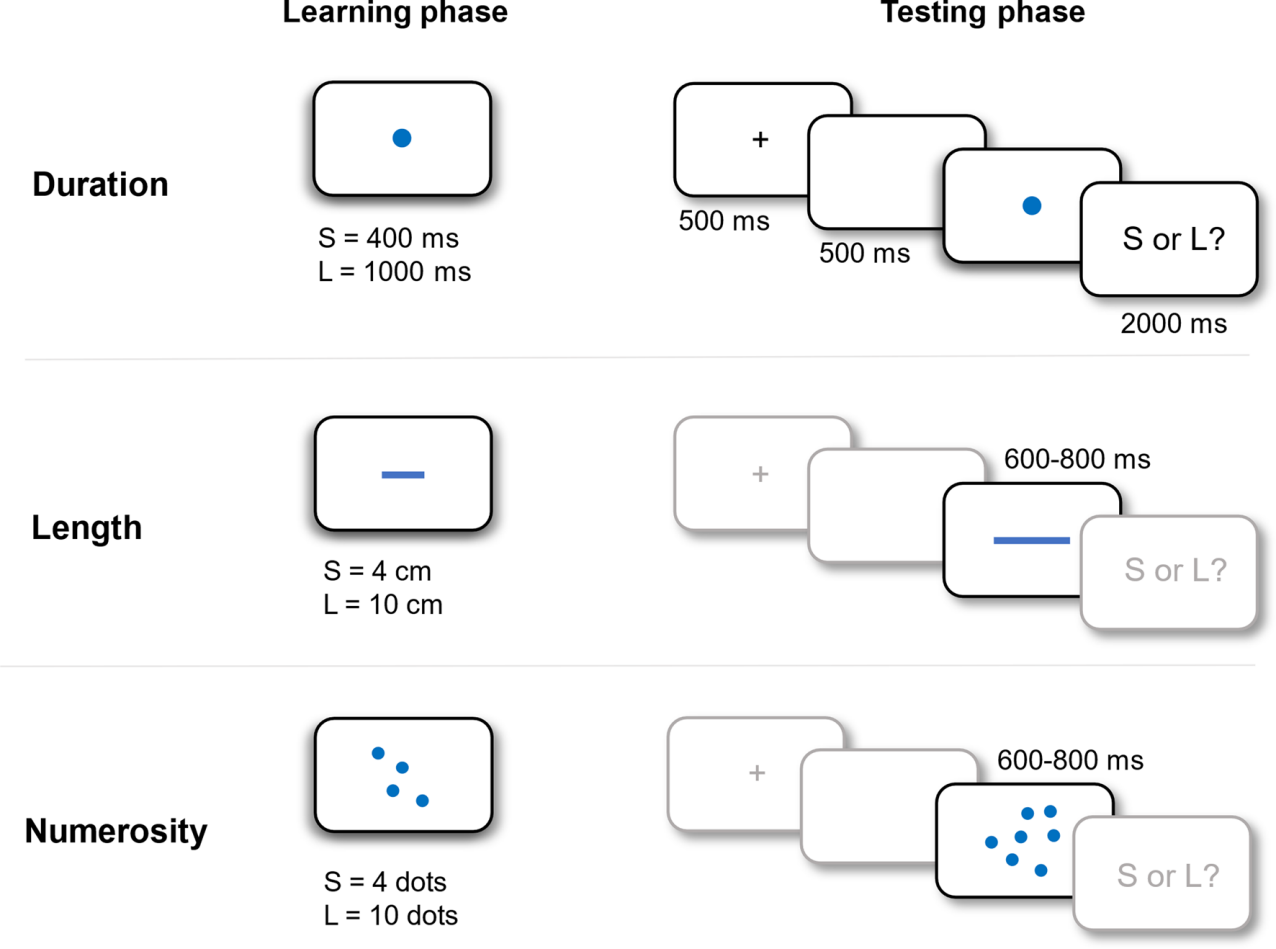
Schematic depiction of the paradigm and of the learning and testing phases of the three bisection tasks

We employed a bisection task to investigate the effect of quantity: duration, length and numerosity (see Droit-Volet 2010 for a similar procedure). Each participant performed three bisection tasks, one task for each quantity (see Fig. 1). The presentation order was counterbalanced between participants. Each task started with a learning phase in which participants were presented with two standards identified at short standard = 400 ms and long standard = 1000 ms for duration, short standard = 4 cm and long standard = 10 cm for length, and few standard = 4 dots and many standard = 10 dots for numerosity. Each standard was presented 10 times for each quantity. No feedback was provided during the learning phase. After the learning phase, participants performed a practice phase in which they familiarized with the task (one practice phase for each quantity, one repetition for each magnitude). During the learning and the practice phase, the experimenter ensured that the instruction and the procedure was fully understood by the participants.

During the testing phase, participants were exposed to new stimuli and instructed to judge if the quantity presented was similar to the standard short/few or the standard long/many by pressing two keys on the keyboard (S = short/few and L = long/many, 2 s response time). Response keys were counterbalanced between participants. Each trial started with a fixation cross lasting 500 ms followed by 500 ms blank screen and then the stimulus (duration, numerosity or length). For each quantity, we presented seven test magnitude levels. For the duration quantity, the stimulus was a blue circle (2.5 cm in diameter) presented for 400, 500, 600, 700, 800, 900 and 1000 ms. For the number quantity, the stimulus was a pattern of 4, 5, 6, 7, 8, 9, and 10 blue dots, each 1 cm in diameter, the spatial disposition of which was pseudo-randomly determined. Finally, for the length quantity, the stimulus was a blue line of 4, 5, 6, 7, 8, 9, and 10 cm (0.5 cm in width). The number and the length quantity were presented for a randomly chosen duration between 600 and 800 s. For each modality condition, the bisection test consisted of two blocks of 42 trials each (six repetitions of each stimulus).

#### Procedure

Participants were individually tested in one experimental session that lasted approximately 45 min. All participants had a normal or corrected-to-normal vision, and all signed informed consent before participation in accordance with the Declaration of Helsinki. The experiment was approved by the Ethics Committee of the Department of General Psychology at the University of Padova (protocol n. 4543).

#### Statistical analyses

Analyses were performed on all 68 participants, discarding trials with missing responses (Young: 4 trials (0.001%) in the duration task, 3 (0.001%) in the length task and 3 (0.001%) in the numerosity task. Elderly: 42 trials in the duration task (0.014%), 23 in the length task (0.008%) and 29 in the numerosity task (0.010%)). Differences in performance between the three quantities and between the two age groups were assessed by means of a generalized mixed-effect model (GLMM) with probit link function, using the *glmer* function of the *lme4* library (Bates et al. 2015) in R (http://www.R-project.org/). Mixed models have several advantages: apart from utilizing individual trial data instead of averaged data, they do not assume independence among observations. The model fitting process considers the covariance structure of the data, incorporating random effects (i.e. individual variability). Also, this methodology has been widely used in clinical studies analysing clinical data (Blini et al. 2016). After standardizing magnitudes within each quantity, we modelled the probability of responding L (long/many) as a function of test magnitude, quantity (duration, length and numerosity), group (young, elderly) and their interactions as fixed-effects, and including by-subject random intercepts and slopes for magnitude and quantity, as well as their interaction, to account for individual variability.

After evaluating the effects of quantity and group on the psychometric curve through Wald chi-square tests, we discarded non-significant effects and compared the reduced model to the full model through Likelihood ratio tests (*anova* function from the *lme4* library). We then proceeded to describe the effects as estimated by the reduced model and perform post hoc tests with the *emtrends* function of the *emmeans* library. A more comprehensive model comparison procedure and additional details on the selected model are reported in the Supplementary material.

### Results

The full model (*R*^2^_marginal_ = 0.77, *R*^2^_conditional_ = 0.94) revealed a significant effect of magnitude (*χ*^2^(1) = 1061.61, *p* < 0.001), a significant interaction between magnitude and quantity (*χ*^2^(2) = 253.43, *p* < 0.001) and between group and magnitude (*χ*^2^(1) = 40.03, *p* < 0.001). No interaction emerged instead between group and quantity (*χ*^2^(2) = 4.32, *p* = 0.11). The triple interaction between magnitude, quantity and group was also not significant (*χ*^2^(2) = 0.67, *p* = 0.71), so we could not individuate specific group differences in response to different quantities. For ease of interpretation, we therefore estimated a reduced GLMM excluding these interactions and confirmed with a Likelihood ratio test that that the full model did not add explained variance to the reduced model (*χ*^2^(4) = 4.79, *p* = 0.31).

**Fig. 2:**
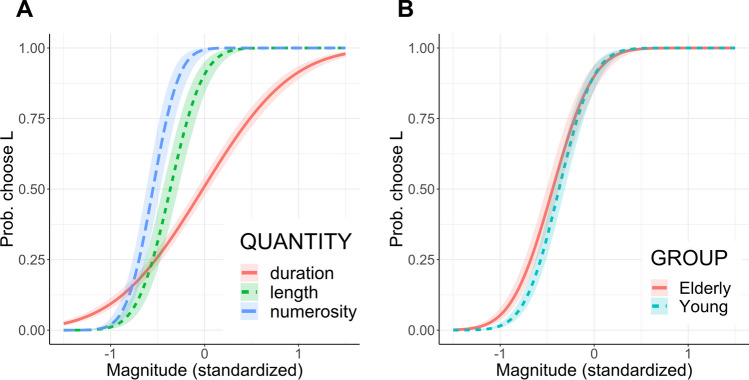
Study 1: modulation of psychometric function from quantity and group. **A** Interaction between magnitude and quantity: lines and 95% CI showing the predicted probabilities of responding L as a function of the magnitude of the test stimulus, separately for each quantity task and averaged across groups. **B** Interaction between magnitude and group: lines and 95% CI showing the predicted probabilities of responding L as a function of the magnitude of the test stimulus, separately for the two groups and averaged across quantity tasks

The reduced model (*R*^2^_marginal_ = 0.76, *R*^2^_conditional_ = 0.94) confirmed a significant interaction between magnitude and quantity (*χ*^2^(2) = 253.04, *p* < 0.001); post hoc tests revealed a flatter psychometric curve in the time bisection task compared to both the length (trend: duration-length = − 2.27, SE = 0.18, z = − 12.81, *p* < 0.001) and number tasks (duration-number = − 3.23, SE = 0.30, z = 10.66, *p* < 0.001), and a shallower slope for length compared to number bisection (length-number = − 0.96, SE = 0.34, z = − 2.86, *p* = 0.01). Predicted responses in the three quantity bisections across young and elderly participants are shown in Fig. 2a. We found a significant interaction also between magnitude and group (*χ*^2^(1) = 35.62, *p* < 0.001), with a steeper curve for young participants compared to elderly ones across all quantity tasks (elderly-young = − 0.56, SE = 0.09, z = − 5.97, *p* < 0.001), as illustrated in Fig. 2b. Since the main effects of quantity (*χ*^2^(2) = 1.14, *p* = 0.56) and group (*χ*^2^(1) = 2.25, *p* = 0.13) were not significant, we could not individuate any difference in under- and overestimation of test magnitude in the two groups or in response to different quantities.

We also computed exploratory correlational analyses to further investigate the relation between tasks in each group (see Supplementary material—correlation analysis).

### Discussion

In Study 1, three bisection tasks were administered to young and elderly groups to investigate the potential changes in magnitude processing. Our results showed that regardless of age, the precision in the temporal discrimination task was lower than in both length and numerical tasks and participants were also less precise in discriminating length compared to numerosity. In addition, healthy elderly participants were overall less precise than young adults.

As previously introduced, past studies provide contradicting results on similarities in sensitivity thresholds in different magnitude domains. In support of a general magnitude system underlying discrimination of different quantities, some studies reported similar discrimination patterns in discrete and continuous quantity, both in spatial and temporal domain (Droit-Volet et al. 2008), or significant correlations within participants between measures of numerical acuity and precision in length discrimination (DeWind and Brannon 2012). In contrast to this interpretation, other research unveiled differences in sensitivity for numerosity, duration and length and in its developmental trajectory (Leibovich and Henik 2014; Odic 2018; Odic et al. 2013). Similarly, in the present study participants showed a higher precision in bisecting discrete quantity compared to continuous magnitudes, and a lower performance in the discrimination of duration compared to length, which suggests an at least partially distinct mechanism for the representation of different quantities, rather than a fully shared magnitude system (Cappelletti et al. 2011; Dormal and Pesenti 2012). However, in line with the idea of a unique discrimination mechanism (Walsh 2003), elderly adults showed a general worse performance in magnitude discrimination: indeed, no domain-specific impairments emerged. Alternatively, as previously suggested, ageing might be responsible for lower sensitivity to duration, numerosity and length as a consequence of a global cognitive decline, affecting for example attention, working memory or other functions important for bisection performance, rather than the underlying representation of a specific quantity or magnitude in general (Lamotte and Droit-Volet 2017).

Age-related modifications have been observed in previous studies focused on a specific domain. With respect to duration discrimination, some authors have explained the age-related temporal decline in terms of modification at the level of the internal clock (Block et al. 1998). However, assuming an age-related slowing of the pacemaker rate, one would expect a shift in the psychometric function in older compared to younger participants, rather than a decrease in discrimination precision as in the present study. Instead, other studies have associated age-related changes in duration processing to reduced cognitive resources required to perform the temporal tasks (Baudouin et al. 2019; Capizzi et al. 2022; Lustig and Meck 2001; Perbal et al. 2002; Turgeon et al. 2016). Notably, ageing is accompanied by reduced processing speed and by a decrease in the rate at which people perform perceptual, motor, and decision-making tasks. Processing speed is a strong predictor of performance across different cognitive tasks in older adults (Salthouse and Ferrer-Caja 2003) and might thus be responsible for age-related cognitive decline also in processing time (Eckert et al. 2010).

The study of numerosity discrimination showed that this ability is resistant to ageing; however, it seems influenced by the decline of other cognitive processes involved in number estimation (e.g. the inhibitory processes) (Cappelletti et al. 2014; Lambrechts et al. 2013). Interestingly, Dormal and colleagues administered both temporal and numerosity tasks to young and older individuals and found ﻿the effect of ageing on duration comparison (i.e. healthy elderly made significantly more errors than young participants), whereas no difference emerged for numerosity comparison (Dormal et al. 2012).

With respect to length processing, no age effect was observed (Lambrechts et al. 2013; Verrillo 1981). However, considering the reduced number of studies conducted, it is difficult to conclude whether length processing is actually not affected by ageing.

Overall, previous research reported heterogeneous results, some of them showing no differences between young and older participants in discrimination tasks, while others showing lower sensitivity in magnitude discrimination during ageing due to a natural cognitive decline in key processes (e.g. attention, memory, speed and inhibition). In the present research, we observed less precision in all discrimination tasks in elderly compared to young participants. Notably, according to our inclusion criteria, only elderly participants with good global cognitive functioning were recruited (participants with a MoCA score lower than 25 were excluded). Despite this rigorous criterion, elderly participants showed a worse performance than younger ones, regardless of the kind of task. All in all, our findings suggest that elderly participants have similar reduction in precision in the processing of different magnitudes and not show domain-specific impairments.

## Study 2

In Study 2, we tested PD patients and healthy controls using the same procedure as in Study 1.

### Method

#### Participants

Thirty older adults with PD and 30 healthy older adults participated in the present study. PD participants were recruited from Parkinson’s disease and movement disorders outpatient clinic of the University Hospital (Palermo, Italy) and Associazione Parkinsoniani (Treviso, Italy). All PD participants had been diagnosed with idiopathic PD by a movement disorders neurologist and evaluated with the revision of the Unified Parkinson’s Disease Rating Scale (UPDRS; Goetz et al. 2008), which indicates the extent of motor disabilities of PD patients. Controls were volunteers from the local community (Padova and Treviso, Italy) and patients’ relatives or friends. All PD patients were tested “on” medication. The MoCA (Santangelo et al. 2015) was used to detect mild cognitive dysfunction. Demographic and clinical characteristics of PD patients and controls are reported in Table 1. Exclusionary criteria for all participants included possible dementia or severe cognitive impairment (as defined by MOCA scores below the cut-off 18), treatment with anticholinergic medications, treatment with certain dopaminergic or benzodiazepine medications known to interfere with cognitive functioning, history of neurosurgery or other neurological conditions, a significant history of or current psychiatric disorders, or any condition which would interfere with testing.

**Table 1:** Descriptive measures (mean and standard deviation) for Parkinson’s patients and healthy older adults

	Parkinson’s patients	Healthy Older adults	*t*	*D*
	n = 30	n = 30		
Age	66.3(9.41)	66.8 (8.00)	0.19	0.05
Education	10.9 (3.80)	14.0 (3.33)	3.39*	0.88
Gender (Male)	16	6		
MoCA	22.4 (4.96)	26.7 (2.27)	3.24*	0.83
MoCA (corrected)	25.0 (3.18)	26.4 (2.47)	1.95	0.50
Years since diagnosis	7.6 (4.38)			
UPDRS	22.3 (6.84)			

*p < .05

MoCA, Montreal Cognitive Assessment; UPDRS, revision of the Unified Parkinson’s Disease Rating Scale

#### Procedure

Participants were tested during an experimental session and performed the same bisection tasks used in Study 1. All participants had a normal or corrected-to-normal vision, and all signed informed consent before participation in accordance with the Declaration of Helsinki. The experiment was approved by the Ethics Committee of the Department of General Psychology at the University of Padova (protocol n. 4543).

#### Statistical analyses

Identical analyses as performed for Study 1 were performed on data from all 60 participants, after discarding trials with missing responses (Elderly: 13 trials (0.005%) in the duration task, 14 (0.006%) in the length task and 17 (0.007%) in the numerosity task. Parkinson: 33 trials (0.013%) in the duration task, 58 (0.023%) in the length task and 22 (0.009%) in the numerosity task). A GLMM with probit link function was used to model the probability of responding L (long/many) including standardized test magnitude, quantity (duration, length and numerosity), group (healthy elderly participants, PD patients) and their interactions as fixed effects. Random effects included by-subject random intercepts and slopes for magnitude and quantity, as well as their interaction. A reduced model was then individuated starting from the full model, by means of model comparison. An additional model comparison procedure is reported in the Supplementary material.

### Results

The full model (*R*^2^_marginal_ = 0.72, *R*^2^_conditional_ = 0.94) highlighted a significant effect of magnitude (*χ*^2^(1) = 423.56, *p* < 0.001), a significant interaction between magnitude and quantity (*χ*^2^(2) = 228.49, *p* < 0.001) and between group and magnitude (*χ*^2^(1) = 13.09, *p* < 0.001), while the triple interaction between magnitude, quantity and group (*χ*^2^(2) = 1.64, *p* = 0.44) and the interaction between group and quantity (*χ*^2^(2) = 1.27, *p* = 0.53) was not significant. A reduced GLMM without these interactions resulted in a similar fit to the data, based on a Likelihood ratio test (*χ*^2^(4) = 2.88, *p* = 0.58).

**Fig. 3:**
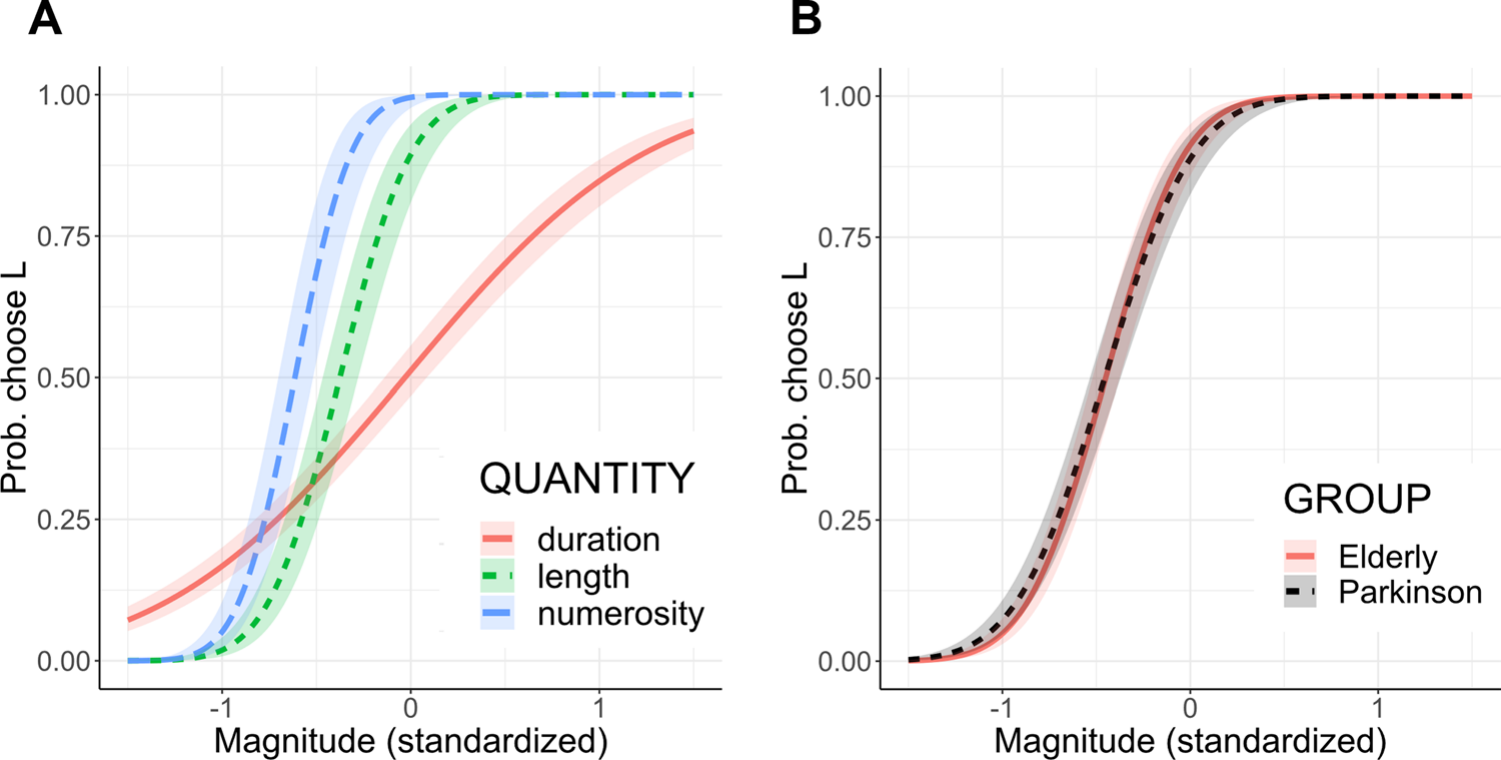
Study 2: modulation of psychometric function from quantity and group. **A** Interaction between magnitude and quantity: lines and 95% CI showing the predicted probabilities of responding L as a function of the magnitude of the test stimulus, separately for each quantity task and averaged across groups. **B** Interaction between magnitude and group: lines and 95% CI showing the predicted probabilities of responding L as a function of the magnitude of the test stimulus, separately for the two groups and averaged across quantity tasks

The reduced model (*R*^2^_marginal_ = 0.72, *R*^2^_conditional_ = 0.94) similarly showed a significant interaction between magnitude and quantity (*χ*^2^(2) = 224.05, *p* < 0.001) and post hoc tests showed also in this sample a steeper slope in the length and number bisection tasks compared to duration judgement (trend: time-length = − 2.33, SE = 0.19, z = − 12.57, *p* < 0.001; time-number = − 3.24, SE = 0.31, z = − 10.49, *p* < 0.001), and a flatter curve for length compared to number bisection (length-number = − 0.91, SE = 0.33, z = − 2.78, *p* = 0.01). These results replicate the pattern found in Study 1. It also emerged a significant interaction between magnitude and group (*χ*^2^(1) = 11.33, *p* < 0.001), revealing a flatter psychometric curve across all quantity tasks for the PD group compared to healthy elders (elderly-PD = 0.34, SE = 0.10, z = 3.37, *p* < 0.001). Predicted responses in the three quantity bisections across both groups and psychometric curves of the two groups across quantity tasks are shown in Fig. 3a-b. Similar to Study 1, we could not detect a difference in the shift of the psychometric curves between groups or between quantity tasks, indicated by the non-significant main effects of quantity (*χ*^2^(2) = 1.00, *p* = 0.61) and group (*χ*^2^(1) = 0.01, *p* = 0.90).

We also computed exploratory correlational analyses to further investigate the relation between tasks in each group (see Supplementary material—correlation analysis).

### Discussion

In Study 2, we investigated the effect of PD on discrimination abilities by comparing healthy older adults and PD patients. As for Study 1, we did not observe interactions between group and quantities, but only their main effects. Again, participants were less accurate in discriminating durations compared to both the numerosity and length tasks, with higher precision for length vs. numerosity bisection. Moreover, PD patients showed overall less precision than healthy controls.

PD has been classically associated with deficits in temporal perception, while other dimensions (i.e. numerosity and space) were poorly investigated in this clinical population. ﻿When compared with healthy controls, PD patients have shown a distortion of perception and reproduction of temporal intervals (O’Boyle et al. 1996; Pastor et al. 1992). Moreover, the more severe the condition, the worse the performance in duration estimation and reproduction tasks (Pastor et al. 1992). The presence of inefficient temporal information processing in PD has been explained by dysfunction in the basal ganglia, a key region for the integration of sensorimotor information (Lucas et al. 2013).

With this background, our expectation was to observe a clear difference in temporal judgements between controls and PD patients. Specifically, a previous study by Dormal and colleagues reported differences between PD patients and young and elderly controls in temporal, but not in numerosity comparisons (Dormal et al. 2012). In particular, the older group made significantly more errors than younger one in the temporal task, and, in addition, PD patients performed slightly worse than healthy elderly participants, suggesting a greater temporal deficit in this clinical population (Dormal et al. 2012). Thus, duration (but not numerosity) processing was affected in both PD and elderly samples, supporting the existence of distinct mechanisms and/or representations for number and time magnitudes (Dormal et al. 2012). In contrast, we did not observe selective temporal deficits in PD, indeed the performance of PD patients did not show specific impairment for the temporal domain, but an overall less precision in the discrimination of all quantities compared to healthy participants.

An aspect to consider is that the paradigm used in our study was different from the one of Dormal et al. (2012), in which participants performed a comparison task instead of a bisection one. In the former case, participants are asked to compare temporal interval trial by trial indicating if the second stimulus last longer than the first, whereas in the latter case participants are asked to first memorize two temporal intervals and then to judge the new temporal intervals with respect to the two previously learned. As far as we know, no previous study has directly compared participants’ performance using these two tasks in a within-subjects procedure, but we can speculate that time discrimination task requires fast decision processing while time bisection task is more demanding on memory processes. If so, we should have observed group differences in our study as well. Notably, other studies exploring the duration processing in PD produced ﻿contradictory results (Jones and Jahanshahi 2014). Limiting to the studies that used the time bisection task, Smith et al. (2007) showed that PD patients (“on” medication) displayed decreased temporal abilities compared to the control group when dealing with longer temporal intervals (1–5 s). However, PD patients performed as accurately as controls when handling shorter temporal intervals (100–500 ms). Merchant et al. (2008) reported increased temporal variability in PD patients compared to controls when dealing with brief temporal intervals (350–1000 ms), but notably, this was observed only when patients were tested off medication. Lastly, Wearden et al. (2008) did not find any indications of temporal impairment in PD patients within the sub-second range (100–800 ms), regardless of whether they were “on” or “off” medication.

Additionally, our task differs also in the presentation mode of numerical information, which was presented sequentially in the paradigm of Dormal and colleagues (2012). However, the dissimilarity in presentation format between quantities should likely have augmented potential differences in discrimination precision, in case of a specifically temporal deficit in PD, rather than hindering it. Our findings highlight that patients with PD are disadvantaged in performing tasks that require the manipulation of different quantities, and this deficit is not limited to the temporal dimension.

## General discussion

The present research investigated how temporal, numerical and length information are processed in healthy and pathological ageing. Our experimental paradigm allowed us to compare the performances in both different discrimination tasks and in different populations (i.e. young vs. healthy elderly and healthy elderly vs. PD patients).

Although some questions remain open, two important results emerged. First, duration judgements were less precise compared to the other magnitudes (both length and numerosity), while the numerosity task reached the higher precision. Second, the elderly and even more PD patients had a worse performance, although no domain-specific impairments were detected. Overall, the first result could be in line with the idea that each quantity is supported by an at least partially independent system.

Due to the nature of the stimuli, it must be noted that the lower precision in duration bisection that emerged in both studies might also be related to the intrinsically sequential nature of temporal magnitude and the necessity to integrate progressively unfolding information compared to numerical and spatial stimuli, which were instead immediately visible to the participants. Nonetheless, the lower performance when judging line length compared to numerosity, both presented statically, suggests that a difference in task difficulty and/or a differential involvement in working memory is not sufficient to fully explain this pattern of result. However, to better clarify the role of presentation mode, future investigations could adopt a dynamic or fully sequential presentation of numerical and spatial stimuli.

On the other hand, healthy elderly and, in particular, PD participants showed a generally (and not selectively) lower precision, which agrees more with the idea of ​​a single processing system for all quantities. However, we cannot exclude that both healthy ageing and PD alter cognitive processes necessary for performing discrimination tasks, rather than magnitude processing per se. While in the second study our sample of PD patients was screened to exclude the potential impact of cognitive dysfunctions, future studies could benefit from considering more specific tests to monitor a potential decline in executive functions also in healthy ageing, to further differentiate between these two hypotheses.

The present study is not exempt from limitations. First, the sample size is quite small and was limited by resource constraints (Lakens 2022). Other studies are therefore required to confirm the observed pattern of results. Second, our conclusions are based on cross-sectional data, which do not allow us to establish causal inference between ageing and discrimination performance. However, this is the first study that compares the ability to discriminate duration, numerosity and length in young healthy, elderly healthy and Parkinson’s disease participants, highlighting interesting differences between groups that should be further investigated in longitudinal research. Future studies should also include additional cognitive measure to investigate the contribution of cognitive resources on temporal, numerical and length in different age groups.

### Supplementary Information

Below is the link to the electronic supplementary material.

Supplementary file1 (DOCX 32 kb)

## Data Availability

All data and scripts are available here: osf.io/8m4kd.
