# Supplementary material for: Duration, numerosity and length processing in healthy ageing and Parkinson’s disease
Source: Eur J Ageing. 2024 Apr 24;21(1):14. doi: 10.1007/s10433-024-00807-z (PMC11043296; doi:10.1007/s10433-024-00807-z)
Supplement: Supplementary file 1 — Supplementary file1 (DOCX 32 kb) [file 10433_2024_807_MOESM1_ESM.docx]

**Duration, numerosity and length processing in healthy ageing and Parkinson’s disease**

Romeo, Z.^1^, Dolfi, S.^2^, D’Amelio, M.^3^, & Mioni, G.^1^

^1^Department of General Psychology, University of Padova, Italy

^2^Department of Developmental Psychology and Socialization, University of Padova, Italy

^3^University of Palermo, Italy

**Supplementary material – Model comparison**

**STUDY 1 (Young vs. Older adults)**

*Model comparison*

In alternative to the hypothesis-driven model definition reported in the main article, we also performed a model comparison to find the model that best describes the data. First, we compared nested models differing only in the random effect structure.

| **GLMM** | **AIC** | **log-likelihood** | **χ^2^ (*df*)** | **p** |
| --- | --- | --- | --- | --- |
| y ~ 1 + (1 \| Id) | 23074.2 | -11535.1 |  |  |
| y ~ 1 + (1 + Magnitude \| Id) | 10731.7 | -5361.8 | 12346.5 (2) | < .001 |
| y ~ 1 + (1 + Magnitude + Quantity \| Id) | 9697.8 | -4837.9 | 1047.8 (7) | < .001 |
| **y ~ 1 + (1 + Magnitude × Quantity \| Id)** | 8567.4 | -4261.7 | 1152.4 (11) | < .001 |

The best model was the null model including a random intercept and random slopes of Magnitude and Quantity as well as their interaction. Based on the selected random effect structure, we assessed the contribution of Magnitude, Quantity, Group and their interaction comparing nested models differing in the fixed effect structure.

| **Fixed effects** | **AIC** | **log-likelihood** | **χ^2^ (*df*)** | **p** |
| --- | --- | --- | --- | --- |
| Null | 8567.4 | -4261.7 |  |  |
| Magnitude | 8500.5 | -4227.2 | 68.94 (1) | < .001 |
| Magnitude + Quantity | 8503.7 | -4226.9 | 0.78 (2) | 0.68 |
| Magnitude × Quantity | 8380.8 | -4163.4 | 126.95 (2) | < .001 |
| Magnitude × Quantity + Group | 8381.3 | -4162.6 | 1.47 (1) | 0.22 |
| **Magnitude × Quantity + Group × Quantity** | 8352.9 | -4147.4 | 30.42 (1) | < .001 |
| Magnitude × Quantity + Group × Quantity  + Magnitude × Group | 8352.8 | -4145.4 | 4.11 (2) | 0.13 |
| Magnitude × Quantity × Group | 8356.1 | -4145.0 | 0.67 (2) | 0.71 |

This procedure reveals that the best model was the model including Magnitude, Quantity, Group and the two-way interactions Magnitude × Quantity and Magnitude × Group, similarly to the reduced model reported in the main article.

| **Parameter** | **Estimate** | **SE** | **z** | **p** | **95% CI** | |
| --- | --- | --- | --- | --- | --- | --- |
| (Intercept) | 0.02 | 0.06 | 0.31 | 0.76 | -0.10 | -0.14 |
| Magnitude | 1.06 | 0.06 | 16.7 | < .001 | 0.94 | 1.19 |
| Quantity (length – duration) | 1.29 | 0.17 | 7.67 | < .001 | 0.96 | 1.63 |
| Quantity (numerosity – duration) | 2.49 | 0.28 | 8.98 | < .001 | 1.95 | 3.03 |
| Group (young – elderly) | 0.01 | 0.09 | 0.15 | 0.88 | -0.16 | 0.18 |
| Magnitude × Quantity (length) | 2.27 | 0.18 | 12.81 | < .001 | 1.92 | 2.62 |
| Magnitude × Quantity (numerosity) | 3.23 | 0.30 | 10.62 | < .001 | 2.63 | 3.82 |
| Magnitude × Group (young) | 0.56 | 0.09 | 5.97 | < .001 | 0.38 | 0.75 |
| **Random effects** | **Variance** | **SD** |  |  |  |  |
| (Intercept) | 0.10 | 0.31 |  |  |  |  |
| Magnitude | 0.10 | 0.31 |  |  |  |  |
| Quantity (length – duration) | 1.53 | 1.24 |  |  |  |  |
| Quantity (numerosity – duration) | 3.74 | 1.93 |  |  |  |  |
| Magnitude × Quantity (length) | 0.72 | 0.85 |  |  |  |  |
| Magnitude × Quantity (numerosity) | 2.85 | 1.69 |  |  |  |  |

**STUDY 2 (Older adults vs. Parkinson’s patients)**

*Model comparison*

As for Study 1, we also performed a model comparison to find the model that best describes the data for Study 2. First, we compared nested models differing only in the random effect structure.

| **GLMM** | **AIC** | **log-likelihood** | **χ^2^ (*df*)** | **p** |
| --- | --- | --- | --- | --- |
| y ~ 1 + (1 \| Id) | 20157.0 | -10076.5 |  |  |
| y ~ 1 + (1 + Magnitude \| Id) | 11245.3 | -5618.7 | 8915.7 (2) | < .001 |
| y ~ 1 + (1 + Magnitude + Quantity \| Id) | 10216.3 | -5097.2 | 1043.0 (7) | < .001 |
| **y ~ 1 + (1 + Magnitude × Quantity \| Id)** | 8744.2 | -4350.1 | 1494.1 (11) | < .001 |

The best model was the null model including a random intercept and random slopes of Magnitude and Quantity as well as their interaction.

As for the comparison of nested models differing in the fixed effect structure and based on the random effect structure described above, the best model converged on the results described in the main article, and included Magnitude, Quantity, Group and the two-way interactions Magnitude × Quantity and Magnitude × Group.

| **Fixed effects** | **AIC** | **log-likelihood** | **χ^2^ (*df*)** | **p** |
| --- | --- | --- | --- | --- |
| Null | 8744.2 | -4350.1 |  |  |
| Magnitude | 8703.5 | -4328.7 | 42.75 (1) | < .001 |
| Magnitude + Quantity | 8706.6 | -4328.3 | 0.89 (2) | 0.64 |
| Magnitude × Quantity | 8602.8 | -4274.4 | 107.80 (2) | < .001 |
| Magnitude × Quantity + Group | 8604.8 | -4274.4 | 0.02 (1) | 0.89 |
| **Magnitude × Quantity + Group × Quantity** | 8596.2 | -4269.1 | 10.60 (1) | 0.001 |
| Magnitude × Quantity + Group × Quantity  + Magnitude × Group | 8598.9 | -4268.4 | 1.29 (2) | 0.52 |
| Magnitude × Quantity × Group | 8601.3 | -4267.7 | 1.58 (2) | 0.45 |

| **Parameter** | **Estimate** | **SE** | **z** | **p** | **95% CI** | |
| --- | --- | --- | --- | --- | --- | --- |
| (Intercept) | 0.10 | 0.08 | 1.24 | 0.21 | -0.06 | 0.26 |
| Magnitude | 1.16 | 0.07 | 15.44 | < .001 | 1.02 | 1.31 |
| Quantity (length – duration) | 1.22 | 0.18 | 6.56 | < .001 | 0.85 | 1.58 |
| Quantity (numerosity – duration) | 2.57 | 0.31 | 8.26 | < .001 | 1.96 | 3.18 |
| Group (Parkinson – elderly) | -0.14 | 0.11 | -1.25 | 0.21 | -0.36 | 0.08 |
| Magnitude × Quantity (length) | 2.33 | 0.18 | 12.57 | < .001 | 1.96 | 2.69 |
| Magnitude × Quantity (numerosity) | 3.23 | 0.31 | 10.49 | < .001 | 2.63 | 3.84 |
| Magnitude × Group (Parkinson) | -0.34 | 0.10 | -3.37 | < .001 | -0.54 | - 0.14 |
| **Random effects** | **Variance** | **SD** |  |  |  |  |
| (Intercept) | 0.16 | 0.40 |  |  |  |  |
| Magnitude | 0.12 | 0.34 |  |  |  |  |
| Quantity (length – duration) | 1.65 | 1.28 |  |  |  |  |
| Quantity (numerosity – duration) | 4.19 | 2.05 |  |  |  |  |
| Magnitude × Quantity (length) | 0.79 | 0.89 |  |  |  |  |
| Magnitude × Quantity (numerosity) | 3.07 | 1.75 |  |  |  |  |

**Supplementary material – Correlation analysis**

**STUDY 1**

*Correlations between tasks*

We explored the correlations between performance measures in the different tasks. We considered as measures of performance the slopes of the psychometrics curves in the three tasks, computed at individual level from the estimated coefficients of the reduced model for both fixed and random effects extracted with the function *coef*(). More specifically:

- SlopeDuration was computed as the sum of the estimated fixed and random effects of Magnitude (since Duration was the reference level of Quantity in the GLMM) and, in case of participants in the Young group, the estimated effect of the interaction Magnitude × Group (young).
- SlopeLength is the sum of the estimated fixed and random effects of Magnitude, the interaction Magnitude × Quantity (length) and, in case of participants in the Young group, the estimated effect of the interaction Magnitude × Group (young).
- SlopeNumerosity is the sum of estimated fixed and random effects of Magnitude, the interaction Magnitude × Quantity (numerosity) and, in case of participants in the Young group, the estimated effect of the interaction Magnitude × Group (young).

Pearson correlations between tasks of the individual slopes are reported in the following table separately for each group. We did not find any significant correlation between performance measures in different quantity bisection tasks, nor within the Young group, nor in the Elderly.

|  | **Young** | | **Elderly** | |
| --- | --- | --- | --- | --- |
|  | **Pearson’s r** | **p** | **Pearson’s r** | **p** |
| SlopeDuration – SlopeLength | - 0.05 | 0.76 | -0.11 | 0.55 |
| SlopeLength – SlopeNumerosity | 0.27 | 0.12 | 0.33 | 0.05 |
| SlopeNumerosity - SlopeDuration | 0.22 | 0.20 | 0.22 | 0.20 |

**STUDY 2**

*Correlations between tasks*

We explored the correlations between performance measures in the different tasks. We considered as measures of performance the slopes of the psychometrics curves in the three tasks, computed at individual level from the estimated coefficients of the reduced model for both fixed and random effects. More specifically:

- SlopeDuration was computed as the sum of the estimated fixed and random effects of Magnitude (since Duration is the reference level of Quantity in the GLMM) and, in case of participants in the PD group, the estimated effect of the interaction Magnitude × Group (Parkinson).
- SlopeLength is the sum of the estimated fixed and random effects of Magnitude, the interaction Magnitude × Quantity (length) and, in case of participants in the PD group, the estimated effect of the interaction Magnitude × Group (Parkinson).
- SlopeNumerosity is the sum of estimated fixed and random effects of Magnitude, the interaction Magnitude × Quantity (numerosity) and, in case of participants in the PD group, the estimated effect of the interaction Magnitude × Group (Parkinson).

Pearson correlations between tasks of the individual slopes are reported in the following table separately for each group. In both Elderly and PD groups, we found a significant correlation between the precision in the duration task and in the length task, as well in the bisection of length and numerosity, while the correlation between numerosity and duration was not statistically significant.

|  | **Elderly** | | **Parkinson** | |
| --- | --- | --- | --- | --- |
|  | **Pearson’s r** | **p** | **Pearson’s r** | **p** |
| SlopeDuration – SlopeLength | 0.60 | < .001 | 0.57 | < .001 |
| SlopeLength – SlopeNumerosity | 0.45 | 0.01 | 0.53 | 0.003 |
| SlopeNumerosity - SlopeDuration | 0.01 | 0.95 | 0.02 | 0.90 |

From these results (Study 1 and 2) we cannot conclude that individuals with a low performance in one quantity bisection task are necessarily the ones showing a large impairment also in all others. However, these analyses should be considered with caution due to the small sample size within each group which might not have enough power to detect the effect of interest.
